# Species-selective plasma metabolism drives lysophosphatidylcholine depletion after cardiac arrest

**DOI:** 10.1016/j.jbc.2026.113536

**Published:** 2026-09-08

**Authors:** Muhammad Shoaib, Jacob S. Kazmi, Mitsuaki Nishikimi, Rishabh C. Choudhary, Jaekyung Cho, Tai Yin, Parmeshar Singh, Cyrus E. Kuschner, Jaewoo Choi, Blanca B. Espin, Lance B. Becker, Li Ma, Junhwan Kim

**Affiliations:** 1 Laboratory for Critical Care Physiology, Feinstein Institutes for Medical Research, Manhasset, New York, USA; 2 Donald and Barbara Zucker School of Medicine at Hofstra/Northwell, Hempstead, New York, USA; 3 Elmezzi Graduate School of Molecular Medicine, Manhasset, New York, USA; 4 Division of Research and Innovation, Oregon State University, Corvallis, Oregon, USA; 5 Department of Emergency Medicine, North Shore University Hospital, Manhasset, New York, USA

**Keywords:** phospholipids, stable isotope, mass spectrometry, metabolism, cardiac arrest

## Abstract

Lysophosphatidylcholine (LPC) is an essential mammalian phospholipid that serves multiple physiological functions. We have previously shown that plasma LPC levels are rapidly reduced after cardiac arrest (CA) in humans and in rodent models, where exogenous supplementation improves survival and neurologic outcomes. However, the specific mechanisms driving this depletion remain undefined, precluding rational design of targeted therapies to preserve LPC levels and limiting clinical translation. Here, we provide the first pharmacokinetic analysis of acute LPC reduction through a novel stable-isotope tracer approach. Our findings demonstrate that saturation status of the fatty acyl chain in LPC species determines the degree of reduction after CA, with polyunsaturated species showing the greatest declines. Further, we found that *in situ* metabolism, not decreased hepatic production nor increased tissue distribution, underlies the observed depletion in plasma LPC after cardiac arrest. Polyunsaturated LPC species showed correlated changes with lysophosphatidic acid and choline-containing metabolites. These correlations likely reflect shared vulnerability to post-CA pathological stress rather than direct metabolic conversion, as conventional metabolic pathways could not account for the temporal patterns and magnitude of LPC reduction, suggesting a broader, systemic dysregulation of plasma lipid metabolism. These findings indicate that therapeutic strategies must focus on either identifying and targeting the novel metabolic mechanisms responsible for species-selective elimination or implementing LPC supplementation protocols designed to overcome accelerated plasma elimination.

Lysophosphatidylcholine (LPC) is an essential phospholipid for physiological cellular function, maintaining membrane integrity, facilitating signal transduction, and supporting energy metabolism (1, 2). While local, acute LPC accumulation has been implicated in cellular toxicity (3), maintaining systemic plasma LPC levels is physiologically critical, with altered plasma levels associated with various pathologies (4, 5, 6, 7, 8, 9). Our previous investigations have revealed that decreased plasma LPC levels are associated with poor neurological and survival outcomes in both human patients and rat models after cardiac arrest (CA) (10, 11, 12). Furthermore, intravenous LPC supplementation improved these outcomes in rat CA models, indicating a crucial role of plasma LPC depletion in post-CA pathophysiology (12, 13).

Despite its demonstrated importance, the precise mechanisms driving LPC depletion after CA remain poorly understood, and similar gaps exist across other conditions in which systemic LPC alterations are observed. Plasma LPC levels are maintained by a complex, dynamic regulation involving both tissue exchange (hepatic release and peripheral tissue uptake) and intraplasma metabolism (*in situ* generation from phosphatidylcholine (PC) and catabolic degradation) (Fig. 1*A*) (1, 14). This intricacy is further complicated by the existence of diverse LPC species, each defined by its specific fatty acid chain (*e.g.*, saturated, monounsaturated, or polyunsaturated), exhibiting distinct synthetic, metabolic, and oxidative pathways (15, 16). To date, no studies have directly examined the metabolic mechanisms driving pathological systemic LPC alterations. Elucidating these species-specific dynamics and their underlying mechanisms is critical not only for understanding LPC's role in CA pathophysiology and developing targeted interventions but also for advancing fundamental knowledge of physiological LPC metabolism that may extend across multiple pathological contexts.

**Figure 1:**
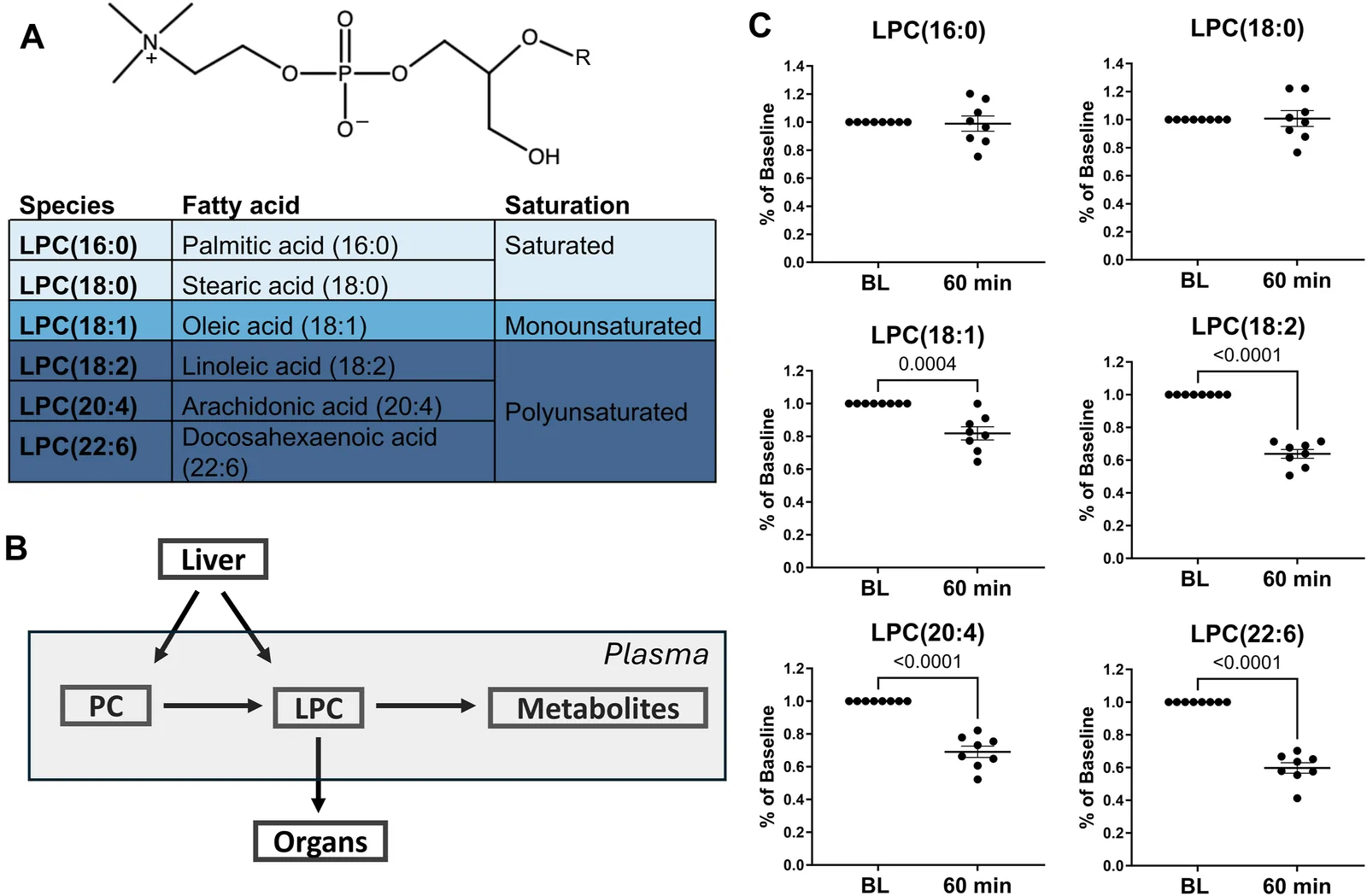
**Species-selective depletion of unsaturated lysophosphatidylcholine (LPC) in plasma after cardiac arrest correlates with fatty acid unsaturation.***A*, schematic of plasma LPC homeostasis, governed by hepatic influx, *in situ* metabolism, and efflux to peripheral organs. *B*, general structure of LPC species, where R represents a single fatty acid chain. Six major circulating mammalian LPC species differ in fatty acyl chain length and degree of unsaturation. *C*, plasma mono- and poly-unsaturated LPC species, but not saturated LPC species, are reduced 60 min after cardiac arrest and resuscitation from baseline in rats, n = 8 per time point. Paired *t* test *versus* baseline.

To address these complexities and determine the mechanisms of LPC depletion after CA, we employed an integrated approach in our rat model. We administered non-natural stable isotope-labeled LPC species (LPC∗) to rats post-return of spontaneous circulation (ROSC) and tracked their plasma decay over time. With no endogenous source of influx for these non-physiological LPC∗, their decay rates directly reflect efflux from plasma, enabling pharmacokinetic quantification of LPC consumption. This kinetic analysis was complemented by empirical measurements of plasma enzyme activities and lipidomic analysis. This integration of tracer kinetics with biochemical profiling uniquely enabled the mechanistic dissection of LPC depletion dynamics, revealing the species-specific drivers of post-CA decline.

Our findings demonstrate that *in situ* LPC metabolism in plasma is the primary driver of post-CA LPC depletion, limiting its availability for tissue repair and contributing to organ dysfunction. This stable isotope tracer approach provides the first direct, real-time kinetic quantification of LPC consumption dynamics in a pathological state, revealing the mechanistic basis for post-CA LPC depletion. These insights advance our understanding of systemic lipid homeostasis under pathological stress and provide the foundation for developing targeted therapies aimed at preserving LPC levels.

## Results

### LPC depletion post-CA is species-specific and correlates with fatty acid unsaturation

Six major LPC species predominate in mammalian plasma: saturated [LPC(16:0), LPC(18:0)], monounsaturated [LPC(18:1)], and polyunsaturated [LPC(18:2), LPC(20:4), LPC(22:6)] species (Fig. 1*B*). We have previously demonstrated species-selective LPC depletion following 10 min of CA, with unsaturated species preferentially affected. To confirm this pattern in our current 12-min CA model and establish the foundation for subsequent mechanistic analyses, we analyzed the levels of these LPC species at 60 min post-ROSC. As expected, LPC species with saturated fatty acid chains, LPC(16:0) and LPC(18:0), remained unchanged (Fig. 1*C*). In contrast, LPC(18:1), containing a monounsaturated fatty acid tail, decreased by 20% at 60 min post-ROSC compared to baseline. LPC species with polyunsaturated fatty acids, LPC(18:2), LPC(20:4), and LPC(22:6), showed more pronounced decreases of 36%, 31%, and 40%, respectively, at 60 min post-ROSC (Fig. 1*C*). These results confirm the correlation between fatty acid unsaturation and the extent of LPC depletion following CA, with polyunsaturated species exhibiting the greatest decline. This reproducible, species-selective depletion pattern provided the basis for investigating the underlying mechanisms driving the preferential loss of unsaturated LPC species.

### Stable isotope tracer analysis reveals accelerated plasma elimination of unsaturated LPC species

To investigate the underlying mechanisms behind the reduction in plasma LPC levels, we injected trace amounts (<4% of endogenous total LPC) of stable isotope-labeled LPC(18:0)∗ (saturated), LPC(18:1)∗ (monounsaturated), and LPC(22:6)∗ (polyunsaturated) species. Experimental scheme and the raw decay of plasma concentrations for each tracer over 60 min post-ROSC is provided in Fig. S1. To quantify elimination kinetics, plasma concentrations of each LPC∗ tracer were plotted on a semi-logarithmic scale (Fig. 2*A*). The corresponding exponential decay equations and R^2^ values are provided in Fig. S1. Notably, at baseline, the elimination rate constants visible in the decay equations followed the hierarchy LPC(22:6)∗ > LPC(18:1)∗ > LPC(18:0)∗, revealing an intrinsic relationship between fatty acyl unsaturation and basal plasma consumption rate. From 6 to 60 min post-injection, concentrations of all three LPC species declined linearly on the semi-log plot, consistent with mono-exponential decay and a one-compartment, first-order elimination model (R^2^ > 0.95 for all species). This linear decay pattern suggests that the initial distribution phase, if present, was completed prior to the first measurement at 6 min and that the mechanisms driving plasma elimination of each LPC species operated consistently throughout the 60-min sampling period. No evidence of a secondary distribution or elimination phase was observed within the measured time window.

**Figure 2:**
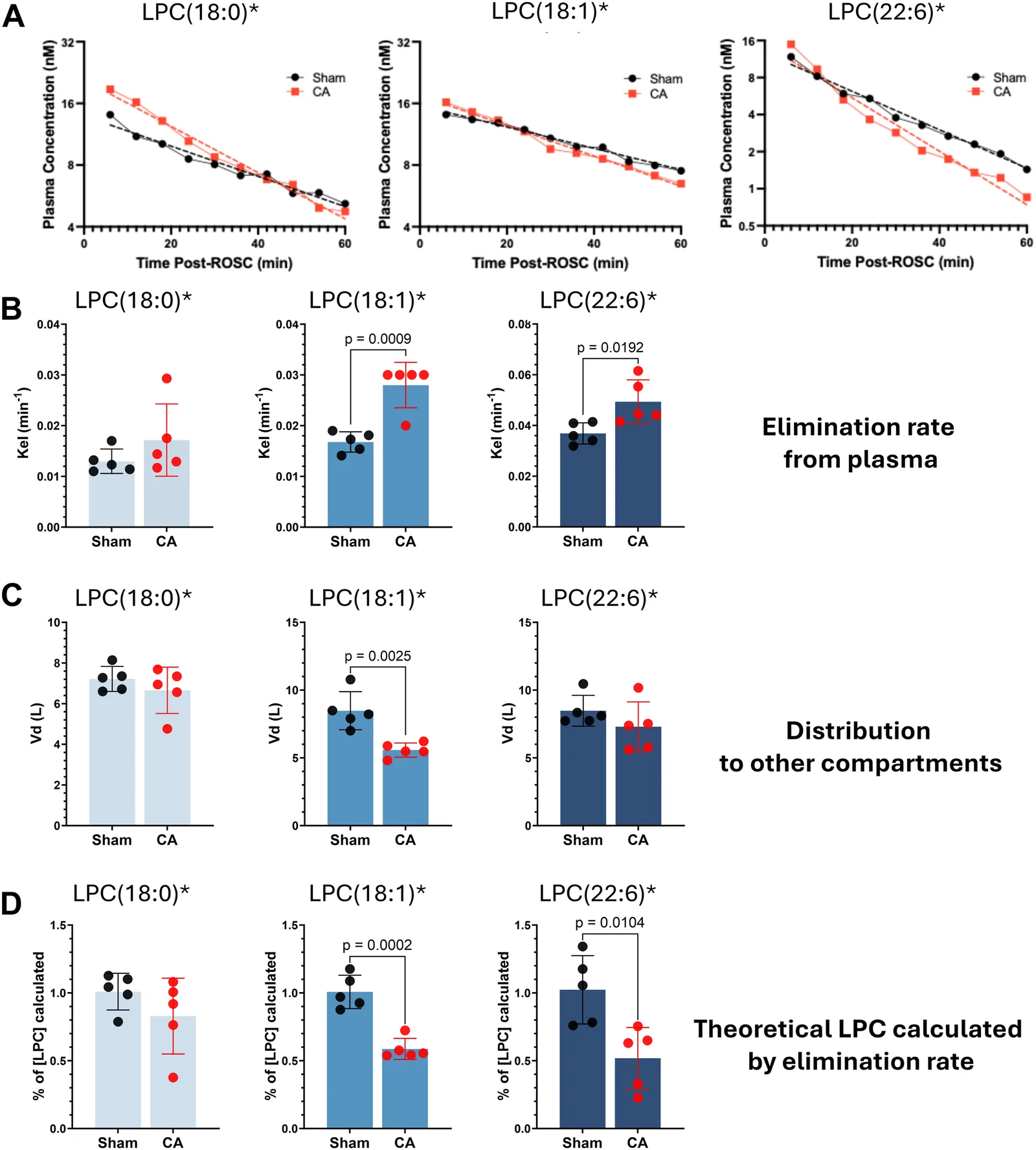
**Stable isotope tracer kinetics reveal accelerated plasma elimination selectively targeting unsaturated LPC species after cardiac arrest.***A*, Semi-logarithmic plot of plasma LPC∗ tracer concentrations over 60 min post-injection in CA and sham-operated rats. All species exhibited linear decay consistent with first-order, one-compartment elimination kinetics (R^2^ > 0.95). *Dashed lines* represent best-fit exponential decay equations. *B*, pharmacokinetic parameters derived from tracer kinetics. CA selectively increased the elimination rate constant (Kel) for unsaturated LPC(18:1)∗ and LPC(22:6)∗, while saturated LPC(18:0)∗ remained unchanged. *C*, volume of distribution (Vd) was significantly decreased for LPC(18:1)∗ and LPC(22:6)∗, while not changed for LPC(18:0)∗. The increased Kel without increased Vd indicates enhanced LPC metabolism within the plasma compartment rather than increased distribution to other compartments. *D*, theoretical plasma concentrations calculated from experimentally determined pharmacokinetic parameters using the exponential decay model. Predicted concentrations at 60 min were 40% lower for LPC(18:1) and 48% lower for LPC(22:6) in CA relative to sham, closely mirroring the empirically observed species-selective depletion pattern in Figure 1, demonstrating that accelerated plasma elimination alone can fully account for post-CA LPC depletion. Values are mean ± SD, n = 5 per group. Mann-Whitney U test.

Pharmacokinetic parameters calculated from the tracer decay show a statistically significant increase in the elimination rate constant (Kel) after CA for LPC(18:1)∗ (53% increase *versus* sham; *p* < 0.001) and LPC(22:6)∗ (32% increase *versus* sham; *p* = 0.019) but not for LPC(18:0)∗ (Fig. 2*B*), indicating that CA selectively accelerates the elimination of unsaturated LPC species from plasma. This finding directly explains the species-specific depletion pattern observed in Figure 1. Consistently, half-lives of LPC(18:1)∗ (41.79 *versus* 27.03 min; *p* < 0.001) and LPC(22:6)∗ (19.01 versus 14.36 min; *p* = 0.011) were significantly reduced in CA compared to sham animals, while the LPC(18:0)∗ half-life remained unchanged (Table S1). The volume of distribution (Vd) for LPC(18:1)∗ (8.48 *versus* 5.57 L; *p* = 0.003) and LPC(22:6)∗ (8.5 *versus* 6.6 L; *p* = 0.035) were significantly reduced in CA *versus* sham animals, while LPC(18:0)∗ showed no significant change (Fig. 2*C*). The combination of increased Kel without increased Vd for unsaturated species indicates that their rapid elimination occurs through enhanced metabolism within the plasma compartment rather than increased distribution to other compartments or sequestration into an unextractable form, either of which would increase Vd. The resistance of LPC(18:0)∗ to these pharmacokinetic changes is consistent with the stability of endogenous LPC(18:0) post-CA observed in Figure 1 and in our previous study (10). Because Vd decreased while Kel increased for unsaturated LPC tracers, these opposing changes offset each other, yielding no significant difference in clearance (Clearance = Kel × Vd) between CA and sham groups (Table S1).

To test whether increased Kel alone could account for the observed LPC depletion, we used the experimentally determined pharmacokinetic parameters to calculate theoretical plasma concentrations over time using the exponential decay model (Fig. 2*D*). Predicted plasma concentrations at 60 min were 40% lower for LPC(18:1) (*p* < 0.001) and 48% lower for LPC(22:6) (*p* = 0.010) compared to sham, closely mirroring the empirically observed depletion patterns in Figure 1, demonstrating that accelerated plasma elimination alone can fully account for the observed LPC depletion. Together, these pharmacokinetic data demonstrate that CA selectively accelerates the plasma elimination of unsaturated LPC species, characterized by increased Kel and decreased Vd, is sufficient to fully account for the observed species-specific LPC depletion pattern, pointing toward enhanced plasma LPC metabolism as the underlying mechanism.

### Tissue phospholipid measurements support plasma-compartment metabolism

To empirically test the pharmacokinetic prediction that accelerated plasma metabolism primarily underlies LPC depletion after CA, we first measured hepatic LPC content and tissue uptake of tracer LPC∗. The liver is a key source of plasma LPC, particularly polyunsaturated species such as LPC(22:6). Our phospholipidomics analysis revealed no significant changes in total or major LPC species in the liver at 60 min post-ROSC compared to sham (Fig. 3*A*). Similarly, there were no significant alterations in total or major PC species (Fig. S2), indicating that hepatic LPC production and release remain stable after ROSC.

**Figure 3:**
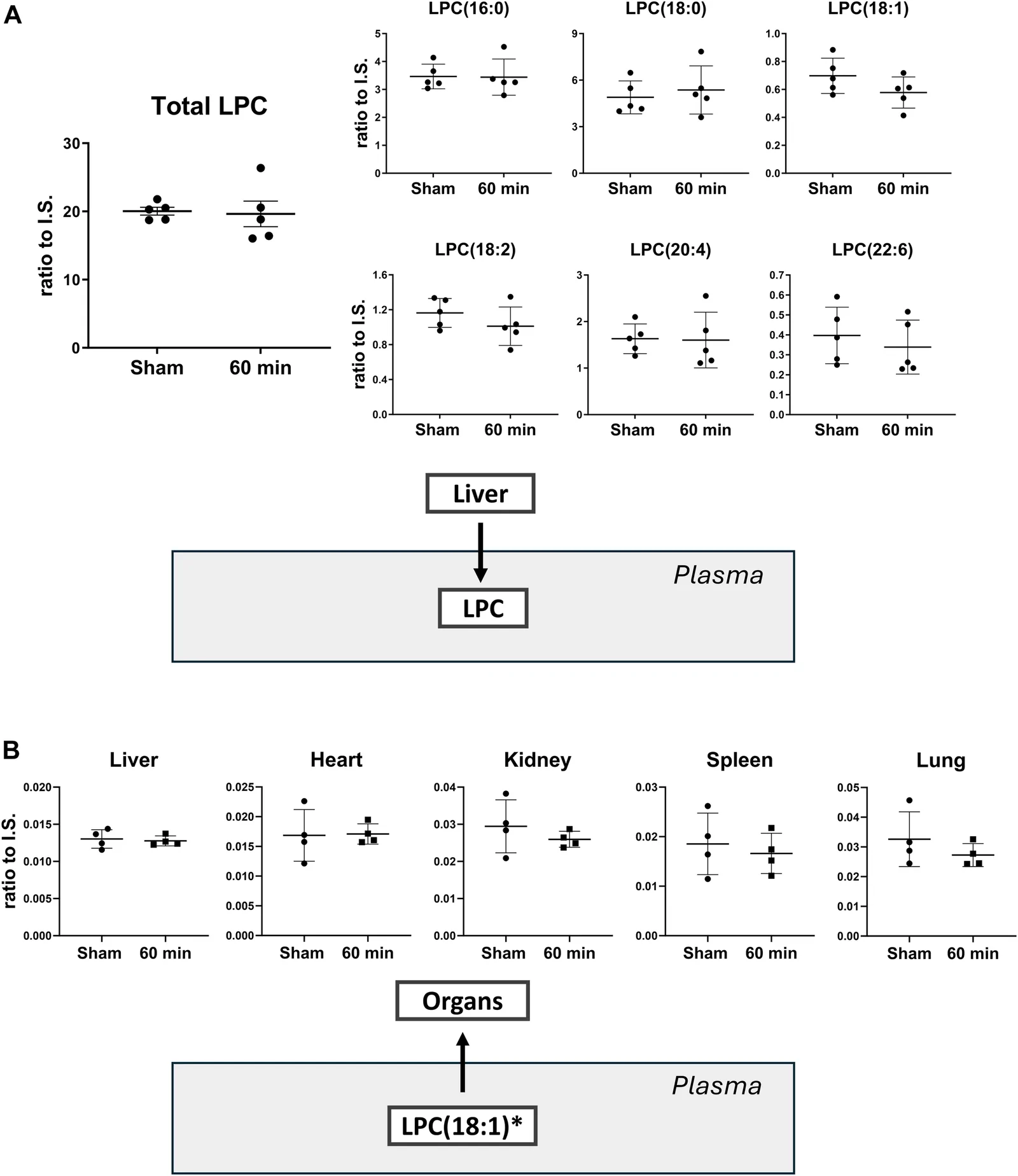
**Hepatic LPC production and peripheral tissue uptake do not contribute to post-CA plasma LPC depletion.***A*, quantification of total and six major LPC species in liver homogenates from CA and sham-operated rats. No significant differences were observed for any species, indicating that hepatic LPC production and release remain unaltered after CA. n = 5 per group, Mann-Whitney U test. *B*, LPC∗ tracer distribution in peripheral organs (liver, heart, kidneys, spleen, and lungs). LPC(18:1)∗ was the only tracer detected in end organs, present in trace amounts with no significant differences between CA and sham groups, indicating no increased tissue uptake after CA. LPC(18:0)∗ and LPC(22:6)∗ were not detected in any tissues examined. Values are mean ± SD. n = 4 per group, Mann–Whitney *U* test.

We also measured labeled LPC∗ species in the heart, liver, kidney, and spleen at 60 min post-injection to assess tissue uptake (Fig. 3*B*). The monounsaturated LPC(18:1)∗ was detected in these organs, and critically, its levels showed no significant changes between sham and post-CA conditions. This lack of difference in tissue LPC(18:1)∗ levels indicates no increased tissue uptake from plasma for this species. Under our experimental conditions, neither the saturated LPC(18:0)∗ nor the polyunsaturated LPC(22:6)∗ were detectable in these organs, and thus increased tissue uptake of these species cannot be entirely excluded. Overall, stable hepatic LPC and PC content and the lack of evidence for increased tissue uptake are consistent with the pharmacokinetic finding that accelerated plasma elimination is the primary driver of the observed decrease in plasma LPC.

### Stable precursor supply and known downstream pathways do not explain species-selective LPC depletion

Having found no evidence of altered hepatic production or tissue uptake as drivers of LPC depletion, we turned to plasma metabolism. To enable direct comparison of LPC with its metabolic precursors and downstream products, we employed multi-class lipidomics to simultaneously detect these metabolites within the same analytical run. We first investigated the most direct and well-known metabolic pathways involving LPC in plasma. Consistent with targeted analysis in Figure 1*C*, only unsaturated LPC species showed significant decreases (Fig. S3), confirming cross-platform reproducibility.

PC serves as the primary precursor for LPC synthesis, primarily through the action of phospholipase A2 (PLA2). Our analysis revealed no significant changes in plasma total PC levels and PC containing PUFAs after CA (Fig. 4). Changes in major individual PC species are shown in Fig. S4. Moreover, there was no alteration in PLA2 activity (Fig. 4), indicating that both the availability of the primary substrate for LPC synthesis and the enzymatic capacity for its production remain stable. These findings suggest that reduced LPC supply is unlikely to be a major contributor to the observed depletion.

**Figure 4:**
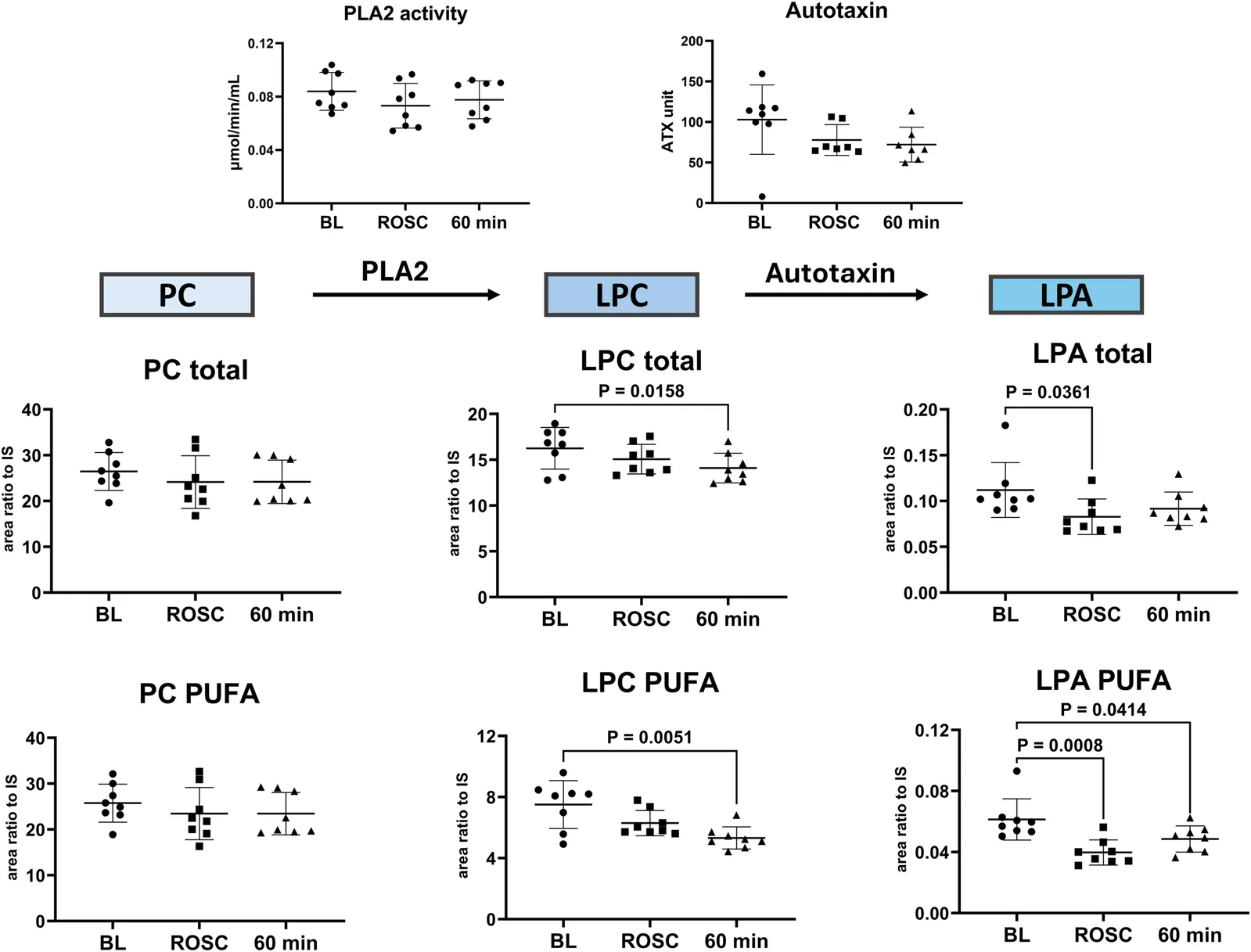
**Canonical LPC metabolic pathways do not account for species-selective depletion.***Top*: Enzymatic activities of PLA2 (converts PC to LPC) and autotaxin (converts LPC to LPA) measured by spectrophotometric assays at baseline, ROSC, and 60 min post-ROSC. PLA2 activity remained stable; ATX showed a decreasing trend. *Bottom*: Plasma levels of total and polyunsaturated (PUFA) species across the PC→LPC→LPA pathway measured by targeted lipidomics. PC (total and PUFA) remained unchanged. LPC (total and PUFA) significantly decreased. LPA (total and PUFA) also decreased, excluding enhanced conversion to LPA as a mechanism of LPC depletion. Values are mean ± SD, n = 8 per timepoint. One-way ANOVA followed by Tukey's multiple comparisons test.

Lysophosphatidic acid (LPA) is a direct metabolic product of LPC, formed by the enzymatic action of autotaxin (ATX) (17). Dysregulation of this pathway is a well-established feature of diverse pathological conditions (18, 19). However, our data showed a slight decrease in total LPA with a decreased trend of ATX activity after CA (Fig. 4). Individual LPA species showed changes that paralleled individual LPC species after CA, with unsaturated species preferentially affected (Fig. S5). This observation indicates that enhanced degradation to LPA may not be a significant mechanism accounting for the substantial decrease in plasma LPC.

We also examined another important downstream metabolic pathway involving choline-containing metabolites, including glycerophosphocholine, phosphorylcholine, and free choline (Fig. 5). Free choline showed a significant transient increase during the early phase of ROSC but returned to baseline levels by 60 min post-ROSC. Phosphorylcholine levels decreased at ROSC and remained suppressed at 60 min. Notably, these changes in choline metabolites did not align temporally with the sustained and gradual decrease in plasma LPC levels observed over the 60-min period.

**Figure 5:**
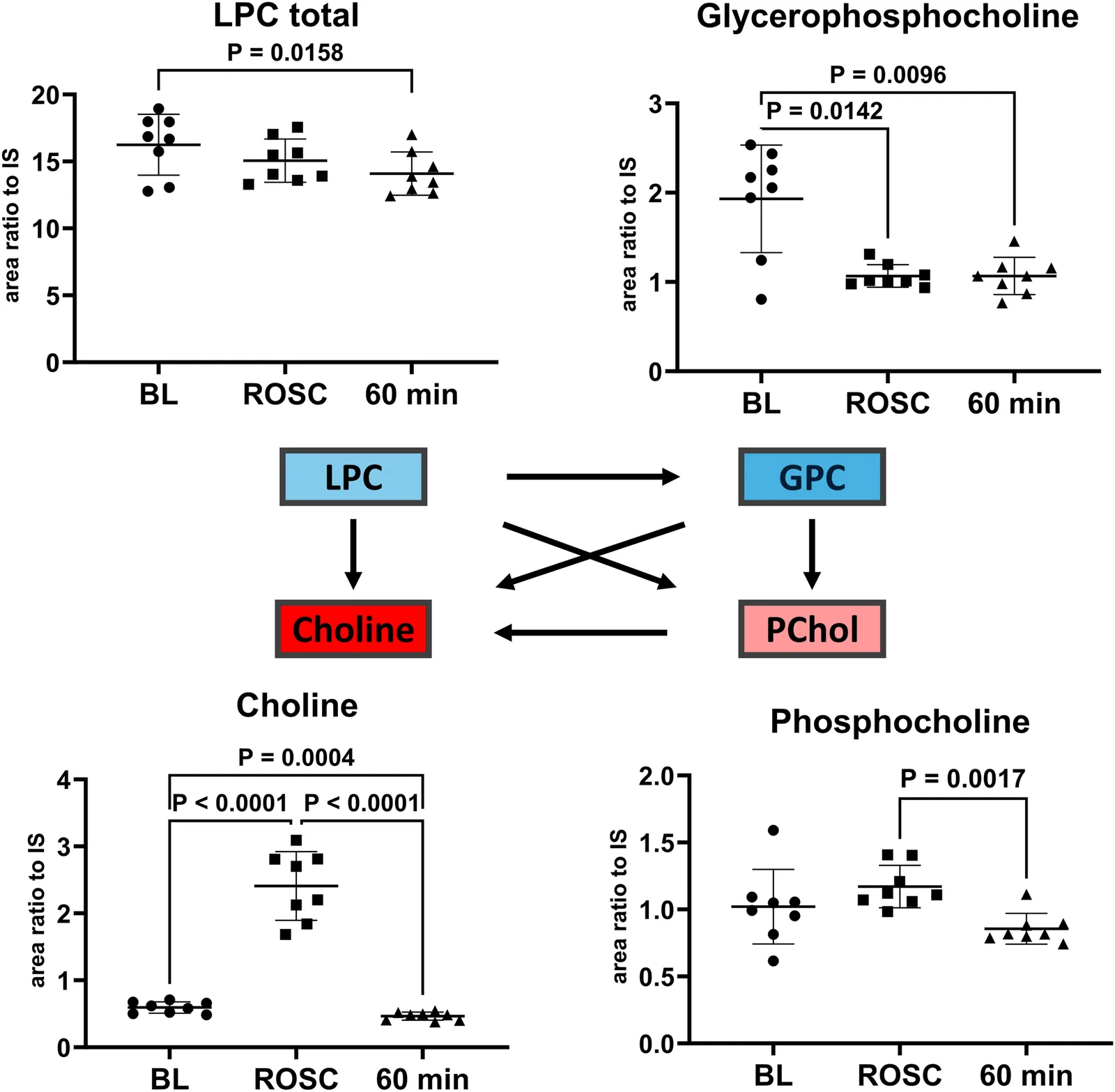
**Choline metabolite patterns are inconsistent with LPC depletion kinetics.** Temporal changes in glycerophosphocholine, phosphorylcholine, and free choline do not align with sustained plasma LPC decline, excluding direct conversion as a mechanism. Values are mean ± SD, n = 8 per timepoint. One-way ANOVA followed by Tukey's multiple comparisons test.

Collectively, the stable PC levels and PLA2 activity are consistent with in-plasma metabolism as the major mechanism; however, the known plasma catabolic pathways examined—LPA/ATX and downstream choline metabolism—do not appear to be the primary drivers of the observed species-selective LPC depletion.

### Correlation analysis reveals shared vulnerability of unsaturated species rather than direct metabolic conversion

To gain mechanistic insights into species-selective LPC depletion, we examined the relationship between individual LPC species and their metabolites. Heatmap visualization showed temporal changes in individual LPC species and individual LPA species and downstream choline metabolites (Fig. 6*A*), revealing that polyunsaturated species across all lipid classes exhibited coordinated decreases over the 60-min post-ROSC period, while saturated and monounsaturated species showed heterogeneous patterns.

**Figure 6:**
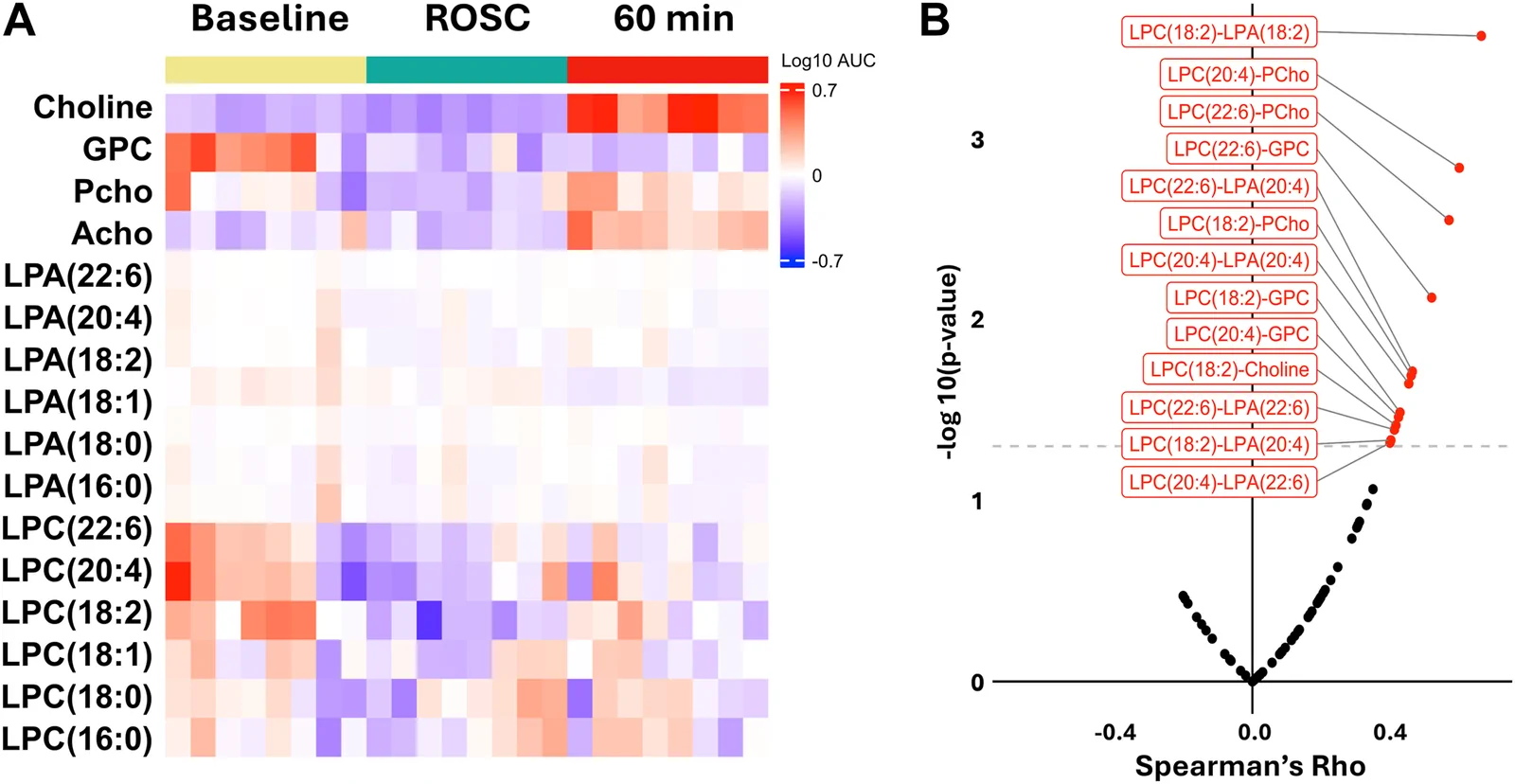
**Correlated changes in polyunsaturated lipid species indicate shared vulnerability.***A*, heatmap of temporal changes in individual species of choline metabolites and individual LPC and LPA species. Color scale shows log10 AUC values: *red* indicates relative increase, *blue* indicates relative decrease. Polyunsaturated LPC species show coordinated decreases across lipid classes. *B*, Spearman correlation volcano plot. *Red dots* = significant pairs (FDR-corrected *p* < 0.05). Strongest correlations: LPC(18:2)-LPA(18:2) (ρ = 0.678), LPC(20:4)-phosphorylcholine (ρ = 0.613), LPC(22:6)-phosphorylcholine (ρ = 0.582), LPC(22:6)-glycerophosphocholine (ρ = 0.531), LPC(22:6)-LPA(20:4) (ρ = 0.474). Saturated species (*black*) showed no correlations. Notably, all significant correlations clustered among unsaturated species, while saturated LPC species (*black dots*, *lower cluster*) showed no significant correlations with any metabolite. These cross-class correlations among unsaturated species, combined with the absence of correlations for saturated species, suggest shared vulnerability to post-CA metabolic stress rather than direct precursor–product relationships. n = 8 per timepoint.

Spearman correlation analysis revealed strong, significant correlations specifically between polyunsaturated LPC species and polyunsaturated LPA species and choline metabolites (Fig. 6*B*). Specifically, the top five correlations were between LPC(18:2) and LPA(18:2) (Spearman's ρ = 0.678, *p* < 0.001), LPC(20:4) and phosphorylcholine (ρ = 0.613, *p* = 0.001), LPC(22:6) and phosphorylcholine (ρ = 0.582, *p* = 0.003), LPC(22:6) and glycerophosphocholine (ρ = 0.531, *p* = 0.008), and LPC(22:6) and LPA(20:4) (ρ = 0.474, *p* = 0.019). By contrast, saturated LPC species showed no such associations. Rather than indicating direct metabolic conversion through any single pathway, these correlated changes across multiple lipid classes suggest a shared vulnerability of unsaturated acyl chains to post-CA pathological stress, implicating complex, multi-pathway perturbations within the plasma compartment.

## Discussion

Circulating LPC plays a critical role in physiological organ function, and its depletion is a recognized phenomenon in various pathological conditions, including neurological disorders, metabolic and inflammatory diseases, aging, CA, and cardiopulmonary conditions (4, 20, 21, 22, 23, 24, 25, 26). Furthermore, the observation that exogenous LPC supplementation ameliorates neurological injury in rat CA models provides causal evidence that LPC depletion actively contributes to post-resuscitation pathology rather than simply reflecting it (10, 11, 12, 13). Despite its established pathophysiological significance, the underlying mechanisms driving this depletion remain unknown. Here, we mechanistically dissect the drivers of species-specific plasma LPC depletion after CA using stable isotope tracer kinetics, enzymatic assays, and targeted lipidomic profiling.

Identifying the mechanisms behind decreased plasma LPC levels presents significant challenges due to its complex metabolic pathways (15, 16). At homeostasis, plasma LPC levels reflect a balance between tissue exchange and intra-plasma metabolism. Tissue exchange includes hepatic secretion and peripheral tissue uptake (1, 15, 27). Within plasma itself, LPC levels are further modulated by *in situ* generation from PC through phospholipase activity and catabolic degradation through multiple pathways (16). This regulation is remarkably complex: Even within the plasma compartment alone, LPC metabolism is intricately integrated into the broader lipidome, where each lipid species has multiple metabolic fates and these pathways are extensively interconnected. These multiple production sources, tissue uptake routes, and complex intra-plasma metabolic transformations, all operating within a vast, dynamic network, make isolating and understanding the precise regulation of any single lipid exceptionally difficult.

To systematically dissect these intricate dynamics, our study employed a novel, multi-stage approach. First, stable isotope tracing allowed us to bypass the confounding variables of production and tissue exchange, revealing that CA selectively increases Kel of unsaturated LPC species while decreasing their Vd—a pharmacokinetic profile consistent with enhanced metabolism within the plasma compartment rather than increased tissue distribution. Furthermore, kinetics modeling demonstrated that this accelerated elimination alone is sufficient to account for the observed species-specific depletion. Second, tissue measurements independently confirmed stable hepatic LPC content and no increased organ uptake, further supporting plasma-compartment metabolism as the major process. Third, targeted analysis of known metabolic routes indicated that this acceleration could not be attributed to the upregulation of any single conventional pathway (*e.g.*, ATX/LPA). Together, these findings identify accelerated intra-plasma metabolism as the dominant pathological process driving LPC depletion, and indicate that it arises from a broader, previously unrecognized dysregulation rather than upregulation of any single conventional pathway.

Our findings reveal broader principles of plasma lipid regulation that extend beyond CA. Specifically, our kinetic analyses under baseline conditions showed that LPC species with higher degrees of unsaturation exhibit intrinsically faster elimination rates despite similar tissue distribution volumes. This demonstrates that plasma lipid composition is not merely a passive reflection of hepatic output and tissue uptake, but is actively shaped by selective, species-specific metabolism within the plasma compartment itself. This principle is highly relevant as LPC depletion is observed across a range of pathological conditions—including stroke, sepsis, diabetes, aging, and neurodegenerative diseases—where underlying mechanisms have remained poorly defined. Our work suggests that these conditions may similarly involve complex, multi-pathway perturbations rather than dysregulation of single metabolic routes. Furthermore, our integrated approach, combining stable isotope tracer kinetics with tissue and plasma pathway analysis, provides a generalizable framework for examining lipid dysregulation across diverse pathological contexts.

These mechanistic insights have direct translational implications for therapeutic strategies targeting LPC depletion. First, pinpointing plasma metabolism—rather than hepatic production or tissue uptake—as the dominant driver of post-CA LPC loss indicates that interventions simply aimed at boosting hepatic LPC output are unlikely to be effective. Instead, rational therapies must focus on stabilizing LPC within the plasma compartment or interrupting the accelerated metabolic processes that preferentially consume unsaturated species. Second, the demonstration that conventional metabolic pathways cannot account for the observed depletion indicates that effective therapeutic interventions will require identifying and targeting the uncharacterized mechanisms responsible for species-selective metabolism. This implies that rational approaches should include (1): direct supplementation of polyunsaturated LPC species with dosing strategies matched to their accelerated elimination kinetics (2); elucidation and therapeutic targeting of the uncharacterized metabolic machinery driving species-selective LPC elimination; and (3) addressing the broader oxidative and inflammatory milieu that may enhance metabolic activity or confer species-selective vulnerability. However, it is important to acknowledge that interspecies differences in lipid metabolism exist between rodents and humans, which may influence the direct translational applicability of these specific therapeutic strategies and warrant careful consideration in future human studies.

Several limitations should be noted. Although the pharmacokinetic analyses were instrumental in guiding our mechanistic understanding, the relatively small sample sizes inherent to these complex kinetic studies necessitate caution when extrapolating the quantitative aspects of these findings. Additionally, while LPC∗ were administered at quantities representing less than 5% of endogenous LPC pools to minimize metabolic perturbation, this exogenous load may nonetheless influence physiological LPC metabolism. Conversely, these deliberately low tracer quantities prevented tracking of downstream metabolic products, limiting direct identification of the specific metabolic pathways responsible for the accelerated elimination. Overcoming this limitation will require higher-sensitivity analytical platforms or higher specific-activity tracers, which would also enable identification of the mechanisms responsible for species-selective LPC metabolism through targeted biochemical fractionation, activity-based profiling, and large-scale lipidomics to capture the full spectrum of plasma metabolic machinery. The present study was designed to identify the mechanism underlying the LPC depletion phenotype previously reported in male rats; extension to female animals, dose-response experiments, and human validation are important future directions. Beyond identifying these mechanisms, elucidating why unsaturation confers metabolic vulnerability—whether through preferential substrate recognition, enhanced oxidative susceptibility, altered physicochemical properties, or combinatorial effects—will provide fundamental mechanistic insight into species-selective metabolism. Finally, determining whether similar plasma-localized metabolic mechanisms operate across other LPC-depleting conditions (stroke, sepsis, neurodegeneration) and are conserved in human patients with CA will establish the broader relevance and translational potential of our findings.

In conclusion, this study delineates the mechanistic basis of plasma LPC depletion following CA, identifying accelerated intra-plasma metabolism of unsaturated LPC species as the principal driver. Through integration of stable isotope tracer kinetics, enzymatic assays, and targeted lipidomic profiling, we demonstrated that this depletion arises independently of hepatic output or tissue uptake, reflecting a plasma-intrinsic disturbance in lipid metabolism. Targeted investigation of known metabolic pathways revealed their inability to account for the observed depletion, instead indicating a broader dysregulation of plasma metabolic networks that selectively targets unsaturated LPC species. This work establishes the first mechanistic framework explaining systemic LPC depletion in a pathological context and reveals a selective vulnerability of unsaturated LPCs to post-ischemic metabolic remodeling.

## Experimental procedures

### Materials

Stable isotope labeled LPC species, LPC(18:0)∗, LPC(18:1)∗, and LPC(22:6)∗, and LPC(17:1), an internal standard, were purchased from Avanti Polar Lipids. 1,2-Dipalmitoyl-sn-glycero-3-phospho-N-methylethanolamine (PME) was purchased from Santa Cruz Biotechnology. Secretory Phospholipase A2 (sPLA2) and lipase activities kits were purchased from Cayman Chemical; lysosomal phospholipase A2 (lPLA2) activity kit, autotaxin activity kit, and lysophosphatidic acid (LPA) concentration kit were purchased from Echelon Biosciences Inc; total choline concentration kit was purchased from Abcam (Cambridge, United Kingdom); and plasma phospholipase C and D activity kits were purchased from Sigma-Aldrich. All other chemicals were purchased from Fischer or Sigma-Aldrich.

### Rat cardiac arrest protocol

All experiments utilized a well-characterized rat model of asphyxial cardiac arrest, and the protocol was approved by the Institutional Animal Care and Use Committee of the Feinstein Institutes of Medical Research (2017-033). Adult male Sprague–Dawley rats (400–500 g, Charles River Production) were maintained under a 12-h light/dark cycle with free access to food and water. The detailed CA procedure is provided in our previous study (16, 28, 29, 30, 31). Briefly, rats were anesthetized with 4% isoflurane, intubated with a 14-gauge catheter, and mechanically ventilated (FiO_2_ 0.3) while anesthesia was maintained at 2% isoflurane. Anesthesia depth was monitored *via* heart rate, blood pressure, and toe-pinch reflex. The left femoral artery and vein were cannulated for arterial pressure monitoring and IV access. After IV heparin (300 U) and vecuronium (2 mg/kg), asphyxial CA was induced by stopping ventilation and isoflurane; CA (MAP <20 mmHg) occurred within 3 min. Following 12 min of asphyxia, ventilation was resumed (FiO_2_ 1.0) and chest compressions (∼300/min) begun. Epinephrine (20 μg/kg IV) was given 30 s after compressions started; ROSC was typically achieved within 1 min with >90% success. Animals that did not achieve ROSC were excluded from the study. Blood samples were collected from the femoral artery at baseline and at predetermined time points after ROSC as specified by each experimental design.

### LPC preparation and administration

LPC(18:0)∗, LPC(18:1)∗, and LPC(22:6)∗ (60 μg each) was suspended in 0.5% bovine serum albumin (BSA) in phosphate buffer saline (PBS; 1X, pH = 7.4) followed by 5 cycles of 10 s sonication. The LPC-BSA mixture was administered within 1 min after achieving ROSC over 1 min time interval to maintain hemodynamic stability. The amount of labeled LPC species was minimal detectable amount to evaluate the rate of decay of labeled LPC species with minimal influence on the physiologic steady state.

### Pharmacokinetic analysis of stable-isotope labeled LPC species

A mixture containing 60 μg each of stable isotope-labeled LPC 18:0∗, LPC 18:1∗, and LPC 22:6∗ was administered intravenously to sham or 12-min CA rats after ROSC (Fig. S1). Blood samples were collected at 6-min intervals post-injection. The measured plasma LPC∗ concentrations were plotted on a semi-logarithmic scale and exponential regression was performed to model the decline over time. Plasma concentration over time was described by the equation: Cp(t) = Cp_0_ · e^(–Kel·t)^, where Cp(t) is the plasma concentration at time t, Cp_0_ is the initial plasma concentration at time zero, and Kel is the elimination rate constant. Pharmacokinetic parameters, such as Kel, half-life (t^1/2^), Vd, clearance (CL_p_), and Area Under the Curve (AUC6-60), were calculated using previously established methods (32, 33). Due to the significantly rapid decrease in plasma concentrations of labeled LPC species and the first time point of blood draw being 6 min after administration, only the elimination t^1/2^, elimination Vd, and elimination CL_p_ were able to be calculated. The AUC6-60 for the time at which blood was drawn was calculated using the trapezoidal rule-extrapolation method.

### Targeted analysis of LPC and PC

Unless otherwise specified, all LPC and PC data in this study were obtained using our established normal-phase HPLC-MS method as previously described (11, 34, 35, 36, 37). Collected blood samples were centrifuged at 1000*g* for 10 min to separate plasma, which was immediately snap-frozen in liquid nitrogen. Harvested tissue samples were blot-dried and pulverized. Lipids were extracted using methanol from plasma and chloroform:methanol (2:1, v/v) from tissue samples, following previously validated methods for quantitative extraction of PC and LPC (36, 38). Lipid extracts were analyzed by normal-phase HPLC coupled with an linear ion trap mass spectrometer (Thermo Scientific) operating in negative ion mode. HPLC conditions and MS parameters were as previously described (36).

### Multi-class analysis of PC, LPC, LPA, and choline metabolites in plasma

To compare LPC, PC, LPA, and choline metabolites, we employed an untargeted lipidomics method as previously described (34). Briefly, plasma samples were extracted in methylene chloride:methanol:isopropanol 25:65:10 (v/v/v) containing 10 μg/ml butylated hydroxytoluene. Ultra-performance LC (UPLC) was performed using a 1.8 μm particle, 100 × 2.1 mm i.d. HSS T3 column (Waters) coupled to a quadrupole time-of-flight (TOF) mass spectrometer (AB SCIEX, TripleTOF 5600) operated in information-dependent MS/MS acquisition mode. PC, LPC, LPA, and choline metabolites were detected and quantified using negative and positive ion modes. Complete UPLC and MS conditions were as previously described (34).

### Statistical analysis

Data for continuous variables are presented as mean ± standard deviation (SD). Statistical analyses were performed using paired *t* test, Mann-Whitney U test, or one-way ANOVA followed by Tukey’s multiple comparisons test, as appropriate. Spearman correlation analysis of lipidomic data was performed on pareto-scaled area under the curve (AUC) values normalized to internal standards, with false discovery rate (FDR) correction applied; no missing values were present in the dataset. Statistical significance was set at *p* < 0.05. GraphPad Prism version 8.4 (GraphPad Software Inc) was used for statistical analysis.

## Data availability

Lipidomic datasets have been deposited in the Metabolomics Workbench database (www.metabolomicsworkbench.org) under Study ID ST005086. All other data are incorporated into the article. Constructs and data sets presented in this study are available upon request.

## Supporting information

This article contains supporting information.

## Conflict of interest

The authors declare the following financial interests/personal relationships which may be considered as potential competing interests:

J. K. has filed a patent application entitled "Lysophosphatidylcholine combinations as a therapy to treat cardiac arrest”. All other authors declare that they have no conflicts of interest with the contents of this article.
