# Supplementary material for: Species-selective plasma metabolism drives lysophosphatidylcholine depletion after cardiac arrest
Source: J Biol Chem. 2026 Sep 8;302(10):113536. doi: 10.1016/j.jbc.2026.113536 (PMC13661218; doi:10.1016/j.jbc.2026.113536)
Supplement: Supplementary file 1 [file mmc1.pdf]

Supporting Information for

Species-selective plasma metabolism drives lysophosphatidylcholine depletion after cardiac arrest

Muhammad Shoaib[1,2,†], Jacob S. Kazmi[1,2,†], Mitsuaki Nishikimi[1,], Rishabh C. Choudhary[1], Jaekyung Cho[1], Tai Yin[1], Parmeshar Singh[1,3], Cyrus E. Kuschner[1,2,3], Jaewoo Choi[4], Blanca B. Espin[1], Lance B. Becker[1,2,3,5], Li Ma[3] and Junhwan Kim[1,2,3,5,*]

[†]M.S. and J.S.K. contributed equally to this work

Correspondence to: Junhwan Kim, PhD

Email: jkim46@northwell.edu

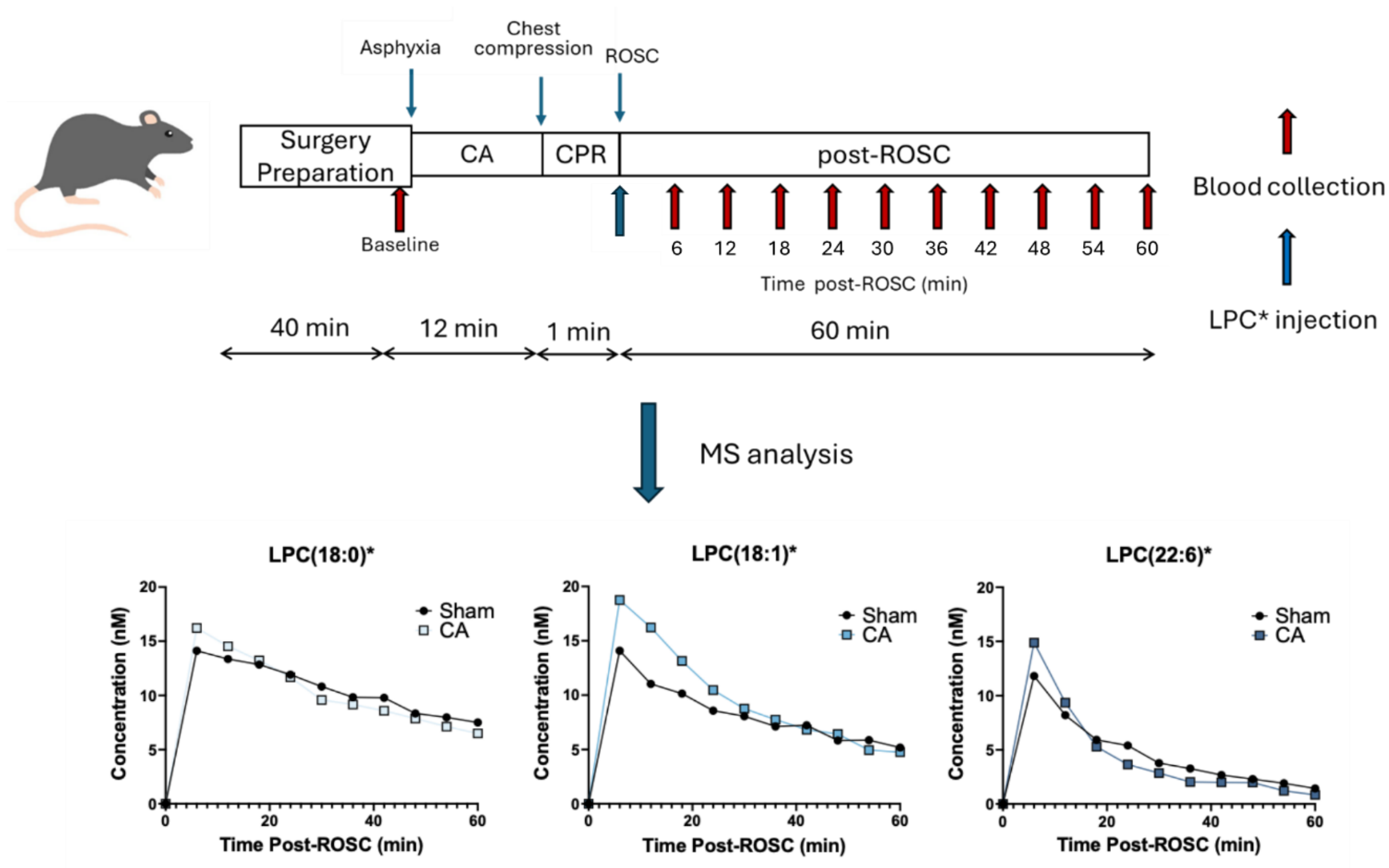

$$Cp(t) = Cp_0 \cdot e^{(-Kel \cdot t)}$$

$$C_{CA(60)} / C_{sham(60)} = e^{-(kel,CA-kel,sham) \cdot 60}$$

	Sham		CA	
	Equation	R^2	Equation	R^2
**LPC(18:0)***	$Y=15.611e^{-0.012X}$	0.9859	$Y=17.503e^{-0.017X}$	0.9866
**LPC(18:1)***	$Y=13.911e^{-0.017X}$	0.9519	$Y=20.795e^{-0.026X}$	0.9860
**LPC(22:6)***	$Y=12.709e^{-0.036X}$	0.9736	$Y=14.888e^{-0.05X}$	0.9521

Supplemental Figure 1. Experimental scheme and raw data from stable isotope tracer analysis. (Top) Timeline of sample collection from rat cardiac arrest model, red arrows represent blood collection timepoints and blue arrows represent LPC* tracer administration. (Middle) Mean traces of absolute LPC concentration after cardiac arrest and resuscitation, n = 5 per group. (Bottom) Exponential decay equations and coefficients of determination for each LPC* species in sham-operated and cardiac arrest rats. ROSC = return of spontaneous circulation, CA = cardiac arrest, CPR = cardiopulmonary resuscitation, MS = mass spectrometry

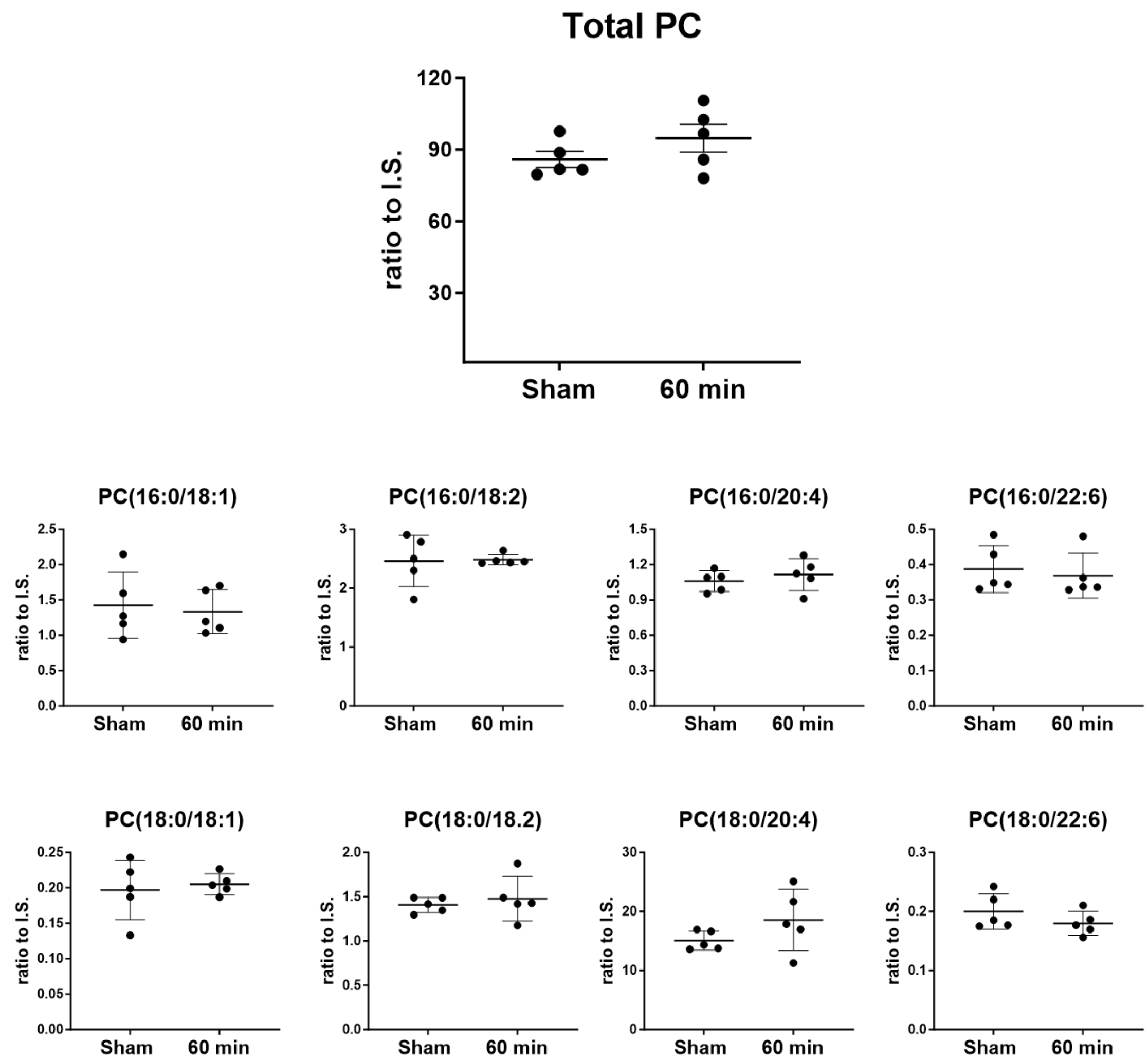

Supplemental Figure 2. Changes in total and major PC species in the liver between sham-operated and cardiac arrest rats. No significant differences were observed for any PC species between groups. Values are mean ± SD, n = 5 per group.

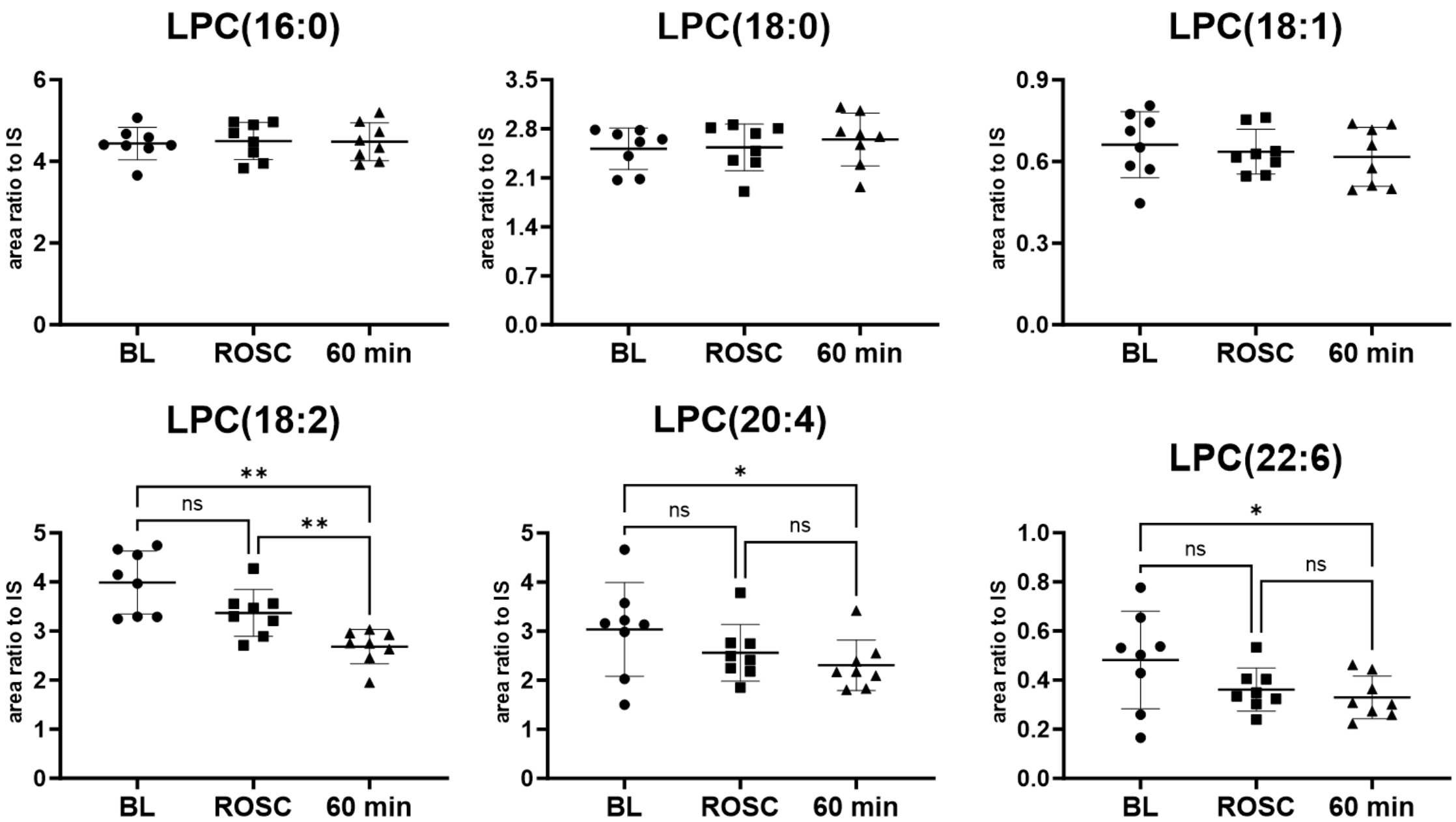

Supplemental Figure 3. Changes in major plasma LPC species after cardiac arrest and resuscitation compared to baseline, measured by untargeted lipidomics. Values are mean ± SD, n = 8 per timepoint. BL = baseline, ROSC = return of spontaneous circulation.

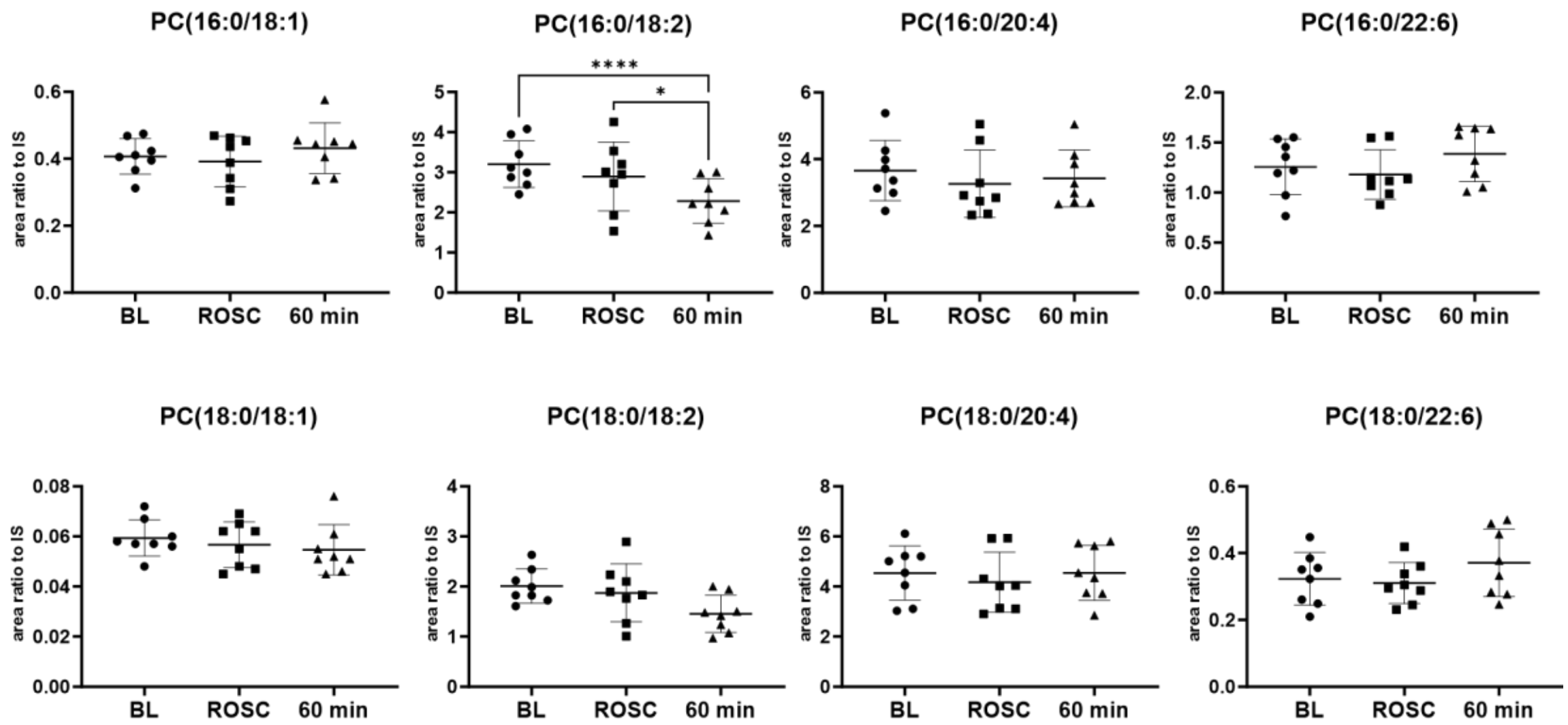

Supplemental Figure 4. Changes in major plasma PC species after cardiac arrest and resuscitation compared to baseline, measured by untargeted lipidomics. Values are mean ± SD, n = 8 per timepoint. BL = baseline, ROSC = return of spontaneous circulation.

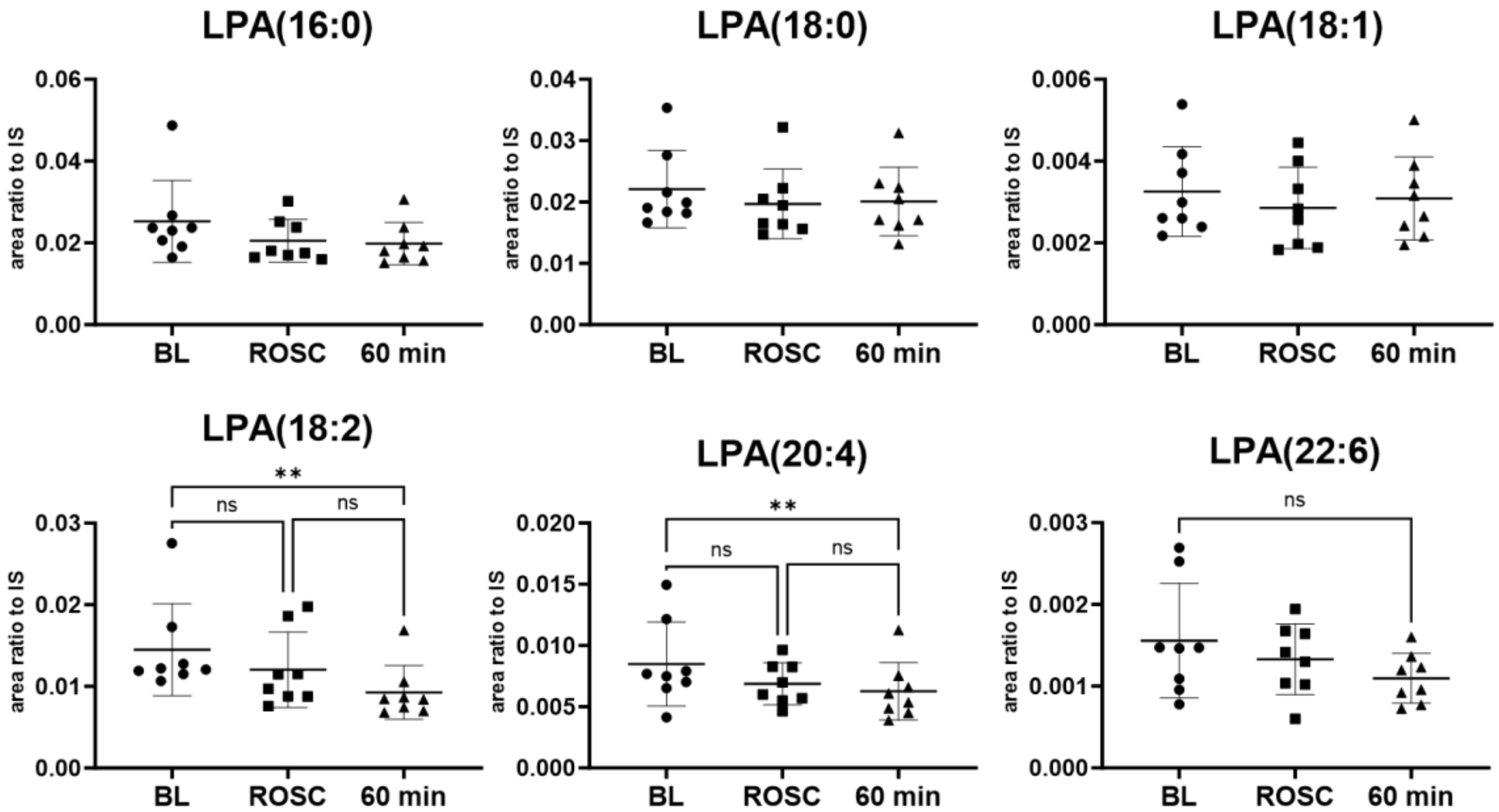

Supplemental Figure 5. Changes in the major LPA species in plasma after cardiac arrest and resuscitation compared to baseline, measured by untargeted lipidomics. Values are mean ± SD, n = 8 per timepoint. BL = baseline, ROSC = return of spontaneous circulation.

Supplemental Table 1. Pharmacokinetic parameters for stable-isotope labeled LPC administration in sham-operated and cardiac arrest rats. Values are mean ± SD, n = 5 per group.

	LPC 18:0*			LPC 18:1*			LPC 22:6*		
	**Sham**	**CA**	***P***	**Sham**	**CA**	***P***	**Sham**	**CA**	***P***
**Volume of Distribution (Vd, L)**	7.2 ± 0.6	6.7 ± 1.1	*0.3602*	8.5 ± 1.4	5.6 ± 0.5	*0.0025*	8.5 ± 1.1	6.6 ± 1	*0.0351*
**Elimination Rate Constant (Kel, min^{-1})**	0.013 ± 0.002	0.017 ± 0.007	*0.249*	0.017 ± 0.002	0.026 ± 0.001	*0.0001*	0.037 ± 0.004	0.049 ± 0.009	*0.0192*
**Elimination Half-life ($t_{1/2}$, min)**	54.7 ± 8.8	44.9 ± 13.9	*0.2186*	41.8 ± 5.2	27.0 ± 2.4	*0.0004*	19.0 ± 2.2	14.4 ± 2.3	*0.0114*
**Elimination Clearance (mL/min)**	93.8 ± 19.6	107.8 ± 20.7	*0.3015*	140.8 ± 16.7	143.1 ± 10.5	*0.8007*	311. ± 47.5	331.3 ± 18.0	*0.4606*
**$AUC_{6\text{-}60}$ (nM·min)**	1261 ± 240	1092 ± 194	*0.2547*	826 ± 98	807 ± 58	*0.7186*	345 ± 52	319 ± 18	*0.3779*
